# Evaluation of Propolis Hydrogel for the Treatment of Dentinal Sensitivity: A Clinical Study

**DOI:** 10.3390/gels9060483

**Published:** 2023-06-12

**Authors:** Saad Mohammed AlQahtani, Raghavendra Reddy Nagate, Manae Musa Musleh Al-Ahmari, Mohammad Al. Magbol, Shankar T. Gokhale, Shreyas Tikare, Saurabh Chaturvedi

**Affiliations:** 1 Department of Periodontics and Community Dental Sciences, College of Dentistry, King Khalid University, Abha 62529, Saudi Arabia or s.alqahtani@kku.edu.sa (S.M.A.); rnagati@kku.edu.sa (R.R.N.); or mamoalahmari@kku.edu.sa (M.M.M.A.-A.); or malmagbol@kku.edu.sa (M.A.M.); sgokhale@kku.edu.sa (S.T.G.); tikane@kku.edu.sa (S.T.); 2 Department of Prosthetic Dentistry, College of Dentistry, King Khalid University, Abha 62529, Saudi Arabia

**Keywords:** dentin hypersensitivity, desensitizers, randomized clinical trial, propolis, hydrogel

## Abstract

Background: Propolis is a natural resinous substance collected by honeybees, chiefly from buds and the leaves, branches, and bark of trees. Its role as a wound-healing gel has been studied, but the use of a propolis hydrogel in the treatment of dentinal hypersensitivity has not been evaluated. Dentin hypersensitivity (DH) is commonly treated via iontophoresis using fluoridated desensitizers. The aim of the present study was to compare and evaluate the effects of a 10% propolis hydrogel, 2% sodium fluoride (NaF), and 1.23% acidulated phosphate fluoride (APF) when used in conjunction with iontophoresis for the treatment of cervical dentin hypersensitivity (DH). Methods: Systemically healthy patients complaining of DH were selected for this single-centre, parallel, double-blind randomized clinical trial. Three substances were selected as desensitizers for study in the present trial: a 10% propolis hydrogel, 2% sodium fluoride, and 1.23% acidulated phosphate fluoride, all in conjunction with iontophoresis. Any decrease in DH following the application of specific stimuli was assessed at baseline, before and after application, on the 14th day following use, and on the 28th day following the intervention. Results: Intra-group comparisons show diminished values of DH at maximum post-op follow-up intervals which were significantly trimmed down from the baseline (*p* < 0.05). The 2% NaF demonstrated a significant reduction in DH over 1.23% APF and the 10% propolis hydrogel (*p* < 0.05). However, there was no statistically significant difference in the mean difference between the APF and propolis hydrogel groups assessed via tactile, cold, and air tests (*p* > 0.05). Conclusion: All three desensitizers have proved to be useful when used in conjugation with iontophoresis. Within the limitations of this study, a 10% propolis hydrogel can be used as a naturally occurring alternative to commercially available fluoridated desensitizers.

## 1. Introduction

Propolis is a naturally occurring resinous, polymeric substance with a complex composition that is collected by honeybees, chiefly from buds and from the leaves, branches, and bark of trees. Sometimes it is also known as “bee glue,” which can be described as a resinous and sticky substance [1]. Nearly 300 different chemical compounds are found in propolis, including polyphenols, amino acids, sesquiterpenes, quinine, coumarins, steroids, and some inorganic materials. Propolis, in contrast to many other natural remedies, has a thorough database of research on its biological activity and toxicity, indicating that it has a wide range of pharmacological activities, including antibacterial, antifungal, antiviral, and anticancer activities, in many traditional texts, including Ayurveda, Chinese medicine, and many more [2]. Several extraction techniques are used to extract the physiologically active parts of propolis. To enrich the active components and therefore boost the pharmacological response, extraction techniques such as traditional maceration and ultrasonic and microwave-aided extraction are applied [3,4,5,6]. With the advancement of biotechnology, propolis can be extracted easily and can be placed in gel bases for varied uses. Its role is well documented in wound healing, but its role in dentistry as a desensitizing agent has not been profoundly evaluated yet.

Dentin hypersensitivity (DH), or dentin sensitivity (DS), is the immediate and painful response of a tooth and is caused by the exposure of the dentinal tubule to the external oral environment [1,2,3,4]. Nevertheless, the Canadian Advisory Board on dentin hypersensitivity considers DH a distinct clinical entity rather than a real pathology. Indeed, thermal, tactile, evaporative, and chemical stimuli are among the methods used to assess the severity of DH [5,6,7,8,9,10,11]. The main three causes of dentine hypersensitivity are (i) recession leading to root exposure, (ii) surface porosity formation followed by the exposure of nascent dentinal tubules, and (iii) pulp nerve sensitivity to changes in fluid movement in the dentine. The explanation for dentinal hypersensitivity that is by far the most widely accepted is the hydrodynamic theory proposed by Brannstrom and colleagues. This theory postulates that fluid movements in the dentinal tubules can activate a baroreceptor and result in neuronal discharge when temperature, physical, or osmotic changes disturb the fluids [12,13].

Many authors have conducted far-reaching research for decades in exploring the definitive treatment for DH. On this journey, many desensitizers have been proposed [14,15]. According to Grossman, desensitizers should have immediate and long-term effects, should be easy to apply and non-irritating, and should not stain teeth. Most proposed desensitizers are salts of sodium or potassium, which are essential ingredients in many types of desensitizing toothpaste currently used. Furthermore, fluorides, chewing gums, lasers, dentin adhesive sealers, and protein precipitators have also been tested; however, none of these options has provided complete relief from DH [14,15].

Propolis is believed to have a favorable effect on pulpal inflammation, caries, and the healing of oral tissues [16,17,18,19,20,21,22] and DH [12,23]. Typically, it is a mixture of 50% resin and vegetable balsam, 30% waxes, 10% essential and aromatic oils, 5% pollen, and 10–15% additional ingredients, such as vitamin A, vitamin B complexes, vitamin E, amino acids, minerals, and other compounds, as well as highly active biochemical substances called bioflavonoids [24,25]. Propolis hydrogels can be made using polyvinyl alcohol (PVA) as a base. PAV has good wound-healing capabilities and can also be of use in the treatment of DH.

Propolis has been shown to reduce dentin hypersensitivity in several in vitro and in vivo studies [25,26]. Almas K et al. observed that propolis is more effective than saline for occluding dentinal tubules [25]. According to the findings of Torwane et al., propolis significantly reduced dentin hypersensitivity [24]. Propolis has also been shown to be effective in reducing the dentin hypersensitivity that results from chairside tooth bleaching. In addition to propolis, other fluoridated desensitizers such as sodium fluoride (NaF) and acidulated phosphate fluoride (APF) are also used for reducing DH. However, the application of these agents alone works in a slow manner, and the resultant relief from DH will be delayed and decreased. In addition to the use of these agents, the use of iontophoresis (a non-invasive method) helps in achieving more effective treatment. The iontophoresis method improves drug delivery through the application of an electric field. It is used in dentistry to create an electro-analgetic impact; it facilitates the ionic movement of the desensitizing material from a high concentration to a low concentration within the dentinal tubules with the help of an electric current, and it blocks the dentinal tubules [27,28,29,30,31]. Fluoridated desensitizers are the most frequently used desensitizers when treating DH with iontophoresis [32,33,34].

The current study aimed to evaluate and compare the efficacy of a 10% propolis hydrogel with 2% sodium fluoride (NaF) and 1.23% acidulated phosphate fluoride (APF) reinforced with iontophoresis for the treatment of DH. According to the author’s knowledge, very little evidence exists on the use of propolis in combination with iontophoresis for DH [35,36,37,38,39,40,41]. Thus, the hypothesis formulated was that a 10% propolis hydrogel reinforced with iontophoresis would provide better relief for DH when compared to iontophoresis-enhanced 2% NaF and 1.23% APF.

## 2. Results and Discussion

A total of 75 patients were recruited for the study and were randomly divided into three groups, with 25 patients in each group. Among the participants, 45 were males and 30 were females, with a mean age of 42.5 years (range of 25–60 years). Of these patients, 40 patients had more than two sites with DH. None of the patients dropped out or were excluded from the study during the follow-up and the analysis of the trial.

Table 1 shows the mean and standard deviation values of the dentin hypersensitivity scale values obtained via the tactile, cold and air tests. The results of the two-way ANOVA show a statistically significant reduction in dentin hypersensitivity scale values within each group, as assessed by the tactile, cold, and air tests from baseline to the 28th-day follow-up (*p* < 0.05) (Table 2).

**Figure 1:**
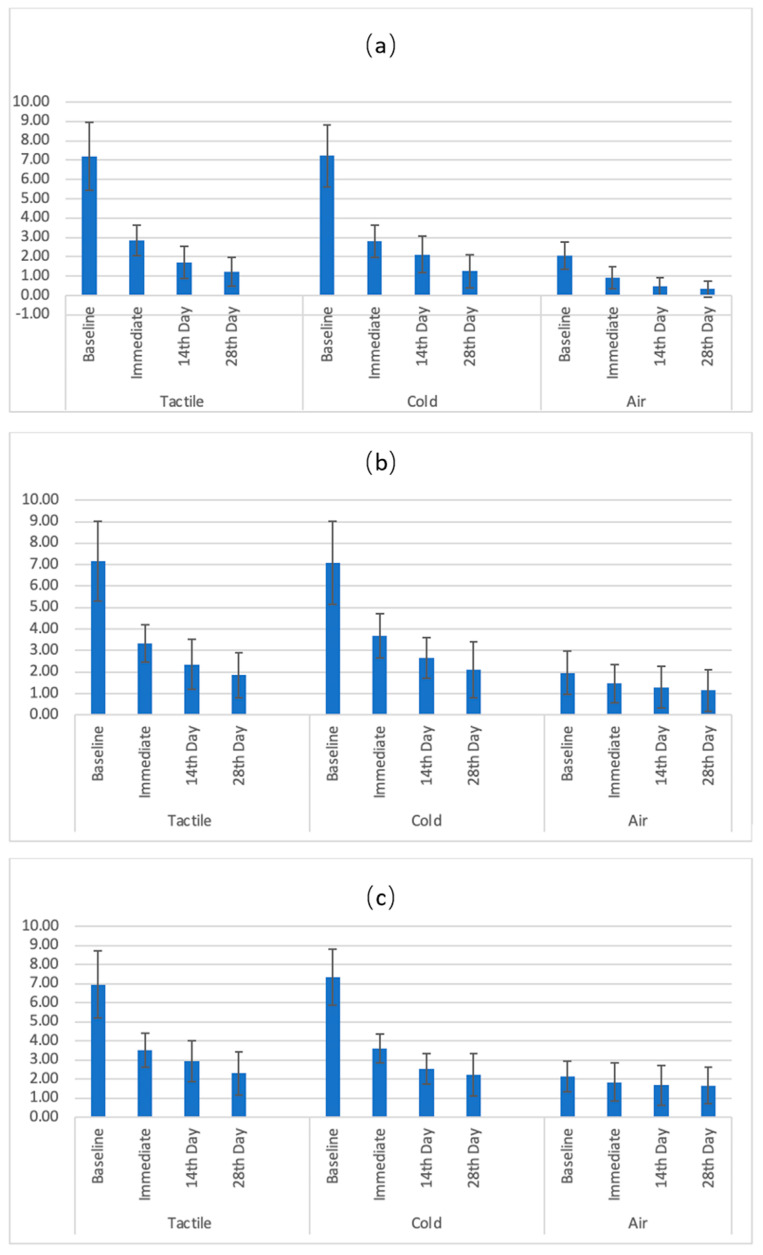
(**a**) Graph showing a comparison of average scores with error bars between different time points (from baseline to 28th-day follow-up) for all assessment methods in the sodium fluoride group; (**b**) graph showing a comparison of average scores with error bars between different time points (from baseline to 28th-day follow-up) for all assessment methods in the acidulated phosphate fluoride group; (**c**) graph showing a comparison of average scores with error bars between different time points (from baseline to 28th-day follow-up) for all assessment methods in the propolis group.

Figure 1a–c graphically show the reductions in mean dentin hypersensitivity scores from baseline to the final follow-up and a comparison of the average scores with error bars from baseline to the 28th-day follow-up for all assessment methods in the sodium fluoride, acidulated phosphate fluoride, and propolis groups. There was a statistically significant difference in the mean DH scores between each agent group (*p* < 0.05) (Table 2). A pairwise comparison of mean difference scores among the agent groups showed that the 2% sodium fluoride group demonstrated a significantly higher reduction in mean scores compared to both the 1.23% acidulated phosphate fluoride and 10% propolis groups, as assessed by different tests (*p* < 0.05). However, there was no statistically significant difference in the mean difference between 1.23% acidulated phosphate fluoride and 10% propolis (*p* > 0.05) (Table 3). Table 4 shows pairwise multiple comparison tests with Bonferroni adjustments of the dentin hypersensitivity scores among the three products assessed via different methods at different follow-up time points (*p* < 0.05).

Despite propolis’ well-known ability to heal wounds [11,12], its medicinal value has received limited attention in dentistry as a desensitizing agent due to several drawbacks [13,14,15,16]. With developments in biotechnology, the use of propolis in a hydrogel form has provided the opportunity to use it as material for the treatment of DH. A hydrogel is a three-dimensional hydrophilic polymeric network with a high absorption capacity for water or biological fluid [17]. The insoluble character of this network structure results from physical and chemical cross-linking, which provide physical integrity. These hydrogel structures inflate in an aqueous environment because of their thermodynamic compatibility. These hydrogels are physically and functionally identical to genuine tissues in terms of their water content, softness, and other characteristics. As a result, hydrogels have demonstrated their value for the delivery of medication and were therefore used in the present study with iontophoresis-reinforced propolis for the treatment of DH.

The treatment of DH remains an enigma to many dentists worldwide. Naturally available and negatively charged flavonoid products such as propolis might occlude dentinal tubules and reduce DH when used with iontophoresis [42,43,44,45]. The superiority of propolis, if any, will be highlighted only when it is compared with other desensitizing agents that have a negative charge and occlude dentinal tubules. The results of this study provide hope to for the use of naturally available agents such as propolis as desensitizers in conjugation with iontophoresis without any adverse reactions. In this study, although propolis did not provide complete relief from DH, our observations encourage multi-directional approaches for DH treatment [46,47,48,49].

Within the author’s knowledge, this is a unique study that evaluates the use of propolis in conjunction with iontophoresis for the treatment of DH and compares the same process with two other fluoride-based desensitizers [50]. This trial was a randomized clinical trial with parallel group assessment. In this study, three different desensitizing agents, 10% propolis hydrogel, 2% NaF, and 1.23% APF, were used in conjugation with iontophoresis to reduce root dentin sensitivity. An assessment of the reduction in DH via various tests plays a crucial role in evaluating different desensitizing agents as there will be variations in the subjective opinions expressed by different individuals [51]. In this study, tactile tests, air blast tests, and cold-water tests were used to check the DH levels at different time intervals.

In our results, iontophoresis with 2% NaF showed a significant reduction in DH (*p* < 0.05). Similar observations can be seen in the previous studies; Brahmbhat et al. [33] compared a 2% NaF solution, hydroxy-ethyl-methacrylate, and glutaraldehyde, (HEMA-G) and NaF-iontophoresis and iontophoresis with distilled water as a placebo. The stimulations used were similar to those used in the current study. Indeed, Brahmbhat et al. concluded that NaF with iontophoresis showed superior results. However, the follow-up periods were longer than in the current study, i.e., three months. Kern et al. [52] observed NaF with iontophoresis for six months and opined its superiority due to the conjugative effects of both. According to these authors, the immediate and long-term impacts might be due to ionic precipitants formed inside the dentinal tubules due to stimulation from iontophoresis. Furthermore, the formation of reparative dentin was also proposed by Arowojolu and Olusile et al., who suggested that it might be the reason for immediate and long-term effects [53].

Similarly, 1.23% APF Iontophoresis also showed a reduction in DH in all time intervals and with all types of stimuli. A study conducted by Aranha et al. [54] showed a decrease in DH only at the ends of three-month intervals, which is wholly different from the current research. The non-usage of iontophoresis might be the reason for the short and immediate effect in this previous study. In another clinical trial by Aparna et al. [55], DH diminution was observed with the use of APF gel in conjugation with iontophoresis, but the time interval of the assessment began on the 14th day of application. The proposed mechanism of action was the formation of fluor-hydroxyapatite crystals on the dentin surface, in addition to the establishment of fluoride salt precipitants inside the dentinal tubules [56].

The 10% propolis hydrogel also showed similar results in the current study that were identical to the previous two agents in all time intervals with all types of stimuli. Madhavan et al. [57] highlighted the superiority of propolis over fluoride gels. However, the proposed active time zone was three months. Again, this might be due to the lack of use of iontophoresis. Torwane et al. [56], who compared 30% Indian propolis with casein phosphopeptide amorphous calcium phosphate (Recaldent™), concluded that Recaldent is superior to propolis. This result agrees with the present study in which NaF was shown to be superior to APF and propolis when used with iontophoresis. However, in the Torwane NA et al. study [56], iontophoresis was not used; therefore, the results are not entirely comparable. According to Sales-Peres et al. [16], the interaction of negatively charged flavonoids with the dentinal tubules and the formation of crystals inside the tubules were proposed to be the background mechanisms for the action of propolis as a desensitizer, which was further ratified by other authors [57,58,59,60,61].

The limitations of this study include that the maximum follow-up time considered in this study was 28 days. Further clinical trials are warranted with more long-term intervals to assess the endurance of the desensitizing effect of propolis. In vitro studies are also needed to assess the long-term pulpal changes and to monitor the exact composition of the dentin-tubule-occluding molecules associated with the newer agent, i.e., propolis. Since the trial was carried out in patients with a wide age range, without gender discrimination, and without site specificity, the results can be applied to a wide range of patients who are suffering from DH.

## 3. Conclusions

Treatment for root dentin sensitivity in addition to ultrasonic scaling will be an essential requirement in the near future. The use of desensitizing agents in combination with iontophoresis can be a valuable option because of their rapid action and sustained effects. Within the limitations of this study, all three desensitizing agents were found to be effective in reducing root dentin sensitivity. Although the propolis hydrogel was not superior to the two other desensitizing agents, can still be used as an alternative to conventional products owing to its availability in nature and its variety of negatively charged flavonoid components. Further long-term trials with much larger samples are needed to achieve a better understanding.

## 4. Materials and Methods

### 4.1. Formulation of the Materials

#### 4.1.1. Preparing 2% Sodium Fluoride and 1.23% Acidulated Phosphate Fluoride

To achieve 500 mL of a 2% sodium fluoride solution, 10. milligrams of sodium fluoride powder was added to 500 mL of distilled water. The acidulated phosphate fluoride was used at a 1.23% concentration and was obtained from the market (APF, Gelato, APF Fluoride Gel, Keystone Industries, Riyadh, Kingdom of Saudi Arabia).

#### 4.1.2. Preparation of 10% Propolis Hydrogel

**Figure 2:**
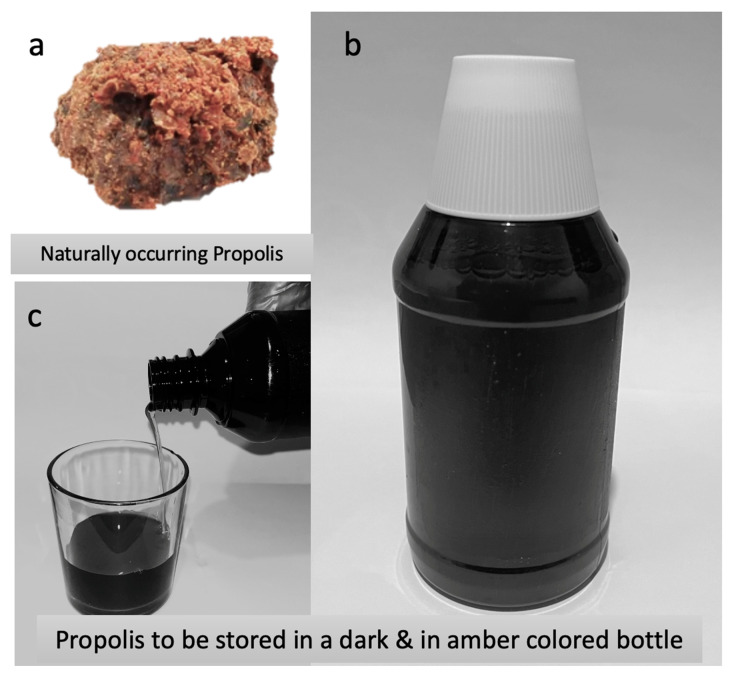
(**a**) Raw, naturally occurring propolis; (**b**) propolis to be stored in an amber-colored bottle; (**c**) propolis after filtration.

Wax-based, raw Indian propolis, which is readily available in stores, was purchased from Hi-Tech Natural Product India Limited in New Delhi, India. According to the procedure outlined by Krell et al., 10% propolis was created after blended the propolis with 70% ethanol and distilled water [62]. The mixture of propolis and ethanol was left in a dark area for 2 weeks. The solution was periodically mixed every day for two weeks. To remove contaminants, the mixture was filtered twice using filter paper from Whatman International Ltd., Maidstone, England. Usually, this mixture of propolis is sustainable and retains its antibacterial effects for over one year. The condition is that it should be kept in the dark. As alcohol-based extracts have longer shelf lives, the ethanol-extracted propolis that was created was preserved without the use of preservatives in a dark, amber-colored bottle (Figure 2). Gel bases were later combined with Indian propolis extract mixture, creating a brown gel that was applied topically throughout the trial.

### 4.2. Study Design

The study protocol was developed, and ethical clearance was obtained from the Institutional Ethics Committee College of Dentistry, King Khalid University, [IRB/KKUCOD/ETH/2018-19/112]. The present trial was designed to follow CONSORT’s (Consolidated Standards of Reporting Trials) guidelines [63]. It was a single-center, parallel, double-blind randomized clinical trial with the registration number NCT05588518. Unrestricted computer-generated randomization was performed centrally. Two blinded examiners conducted the DH measurements, and averages were recorded at pre-decided time intervals. To avoid bias, the examiners were provided with a fresh sheet of paper each time they examined the patients and recorded measurements for the study to conceal the previous measurements. An additional blinded author conducted interventions. For Examiners 1 and 2, Cronbach’s Alpha was found to be 0.92 for inter-examiner reliability and 0.85 and 0.82 for intra-examiner reliability.

### 4.3. Study Participants

**Figure 3:**
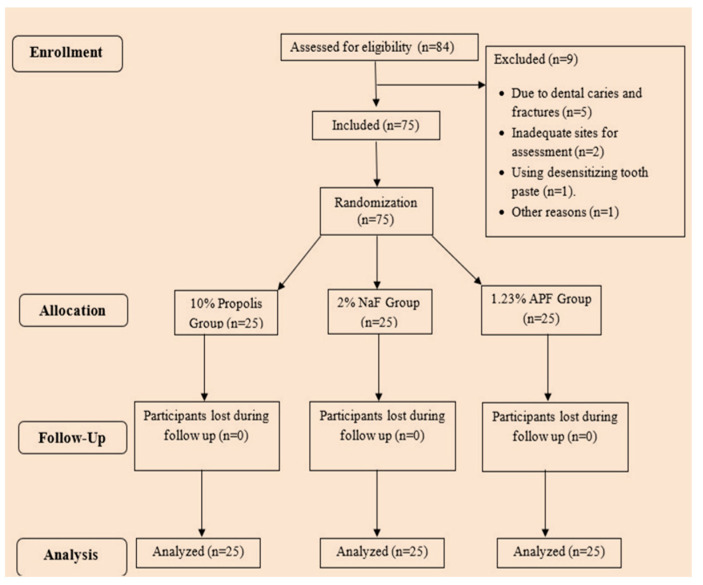
CONSORT flow chart for patient allocation.

Patients reporting to the Department of Periodontics for DH from April 2019 to August 2020 were included in this study. The ages of the patients ranged from 25 to 60 years. The participation was entirely voluntary, and a signed statement of informed consent was obtained from each patient prior to the start of the study. The inclusion criteria were (1) a systemically healthy subject (based on medical and drug histories), (2) a subject requiring scaling and polishing with moderate calculus with at least two non-adjacent hypersensitive teeth with Schiff scale scores >1 with air blast stimulus, and (3) access to at least 10 tested, natural teeth other than the third molars. Patients with dental caries, those undergoing orthodontic treatment, patients with a history of periodontal treatment, patients using desensitizing toothpaste, patients receiving DH treatment, pregnant women, individuals with a history of asthma, and those who were allergic to honeybee products and pollen were excluded from the study. After randomization, the patients were assigned to three test groups. All three groups received oral prophylaxis, after which the initial DH levels were assessed. Figure 3 shows patient allocation as a flow chart.

### 4.4. Application of Desensitizing Agents

Using a cotton pellet, Vaseline was frequently applied all over the gingiva and mucosa to protect the delicate tissues from any negative effects. The 10% propolis, 2% sodium fluoride and 1.23% acidulated phosphate fluoride were the desensitizers utilized in the test groups for the first, second, and third groups, respectively. A pea-sized amount of the appropriate desensitizer was applied to the test tooth. Iontophoresis was then administered immediately to all three groups. The desensitizing agent was administered at two different times: immediately following oral prophylaxis and on the 14th-day visit.

### 4.5. Application of Iontophoresis

This study made use of iontophoresis technology from Jonofluor Scientific, Medical S.R.L., Via Olivera, 42 31020 San Vendemiano [TV], Italy. The process entailed applying an electric current to the test tooth to check for the penetration of ions through the dentinal tubules. The correct tray size was chosen based on the size of the patient’s arch. The desensitizer was placed on the tray and covered with a sponge. The specific desensitizer used in the sponge depended on the relevant subject’s study group. The sponge and tray were then placed in the patient’s mouth. The patient was told to use his or her palm to hold the iontophoresis device’s positive electrode. The metal plate in the tray was connected to the negative electrode. Once the device was turned on, a 2 mA current was applied for one minute, in accordance with the manufacturer’s recommendations. The unit was turned off after one minute, and the opposite arch was then subjected to the same operation. For the next 30 min, the patient was recommended not to consume any liquids, eat anything, or gargle.

### 4.6. Assessing Clinical Parameters

To assess the clinical parameters, the following stimuli were used in the specified order: tactile, air blast, and cold water. The improvement in DH was recorded at baseline immediately after ultrasonic scaling and shortly after the application of the desensitizer, 14 days prior to and after desensitizer application, and 28 days postoperatively without any application. At each interval, the desensitizers were carefully tested for any negative effects. A pressure-sensitive probe (TPS; VIVACARE^®^, Vivadent, Schaan, Liechtenstein) was used to conduct the tactile test. The probe was pushed from the distal to the mesial direction immediately apical to the cementoenamel junction and evaluated on a 1–10 visual analogue scale (VAS) depending on the patient’s discomfort. Using a four-graded Schiff scale, air stimulation was measured. Zero was interpreted as no response, one as a response noted but the subject did not want the stimulus to be stopped, two as a response noted and the subject requested that the stimulus be stopped, and three as pain observed and the subject requested that the stimulus be stopped immediately. A cold-water test was performed, using a disposable syringe to apply cold water drop by drop while measuring the result using VAS. Colgate Regular Toothpaste (Colgate-Palmolive Company India Ltd., Gujarat, India) and a soft-bristle toothbrush were given to each study participant across all study groups. A modified Stillman’s toothbrushing technique was presented to the study participants on a larger tooth model, and they were told to brush their teeth for 2–3 min once in the morning and once before bed each night from the first day until the end of the follow-up period.

### 4.7. Power Calculation and Statistical Analysis

A prior power calculation in the study was carried out based on previous results [34]. The sample size was estimated to be 25 subjects per group, using the formula $n=\frac{2(S^{2})}{d^{2}}{\left[z_{1-\alpha /2}+z_{1-\beta }\right]}^{2}$, to obtain a study power of 85%. Statistical analysis was conducted using SPSS, version 20. The Kolmogorov–Smirnov test showed a normal distribution of the data; hence, parametric tests were used for analysis. Intra-group comparisons were performed with a two-way repeated measures analysis of variance (ANOVA) test. Intergroup comparisons were performed using pairwise post hoc tests with Bonferroni adjustments where *p* < 0.05 was taken as statistically significant.

## Figures and Tables

**Table 1:** Mean dentin hypersensitivity scores of three clinical products (sodium fluoride, acidulated phosphate fluoride, and propolis) assessed via tactile, cold and air tests at different follow-up periods.

Assessment Method	Products	Time Points	N	Mean	SD	SE
Tactile test	Sodium fluoride	Baseline	25	7.20	1.77	0.35
		Immediate	25	2.84	0.79	0.16
		14th Day	25	1.70	0.83	0.17
		28th Day	25	1.22	0.75	0.15
	Acidulated phosphate fluoride	Baseline	25	7.18	1.85	0.37
		Immediate	25	3.34	0.86	0.17
		14th Day	25	2.36	1.17	0.23
		28th Day	25	1.86	1.06	0.21
	Propolis	Baseline	25	6.96	1.77	0.35
		Immediate	25	3.52	0.88	0.18
		14th Day	25	2.94	1.06	0.21
		28th Day	25	2.30	1.12	0.22
Cold test	Sodium fluoride	Baseline	25	7.22	1.60	0.32
		Immediate	25	2.80	0.83	0.17
		14th Day	25	2.12	0.95	0.19
		28th Day	25	1.26	0.87	0.17
	Acidulated phosphate fluoride	Baseline	25	7.10	1.94	0.39
		Immediate	25	3.68	1.04	0.21
		14th Day	25	2.66	0.94	0.19
		28th Day	25	2.10	1.31	0.26
	Propolis	Baseline	25	7.34	1.48	0.30
		Immediate	25	3.62	0.75	0.15
		14th Day	25	2.54	0.82	0.16
		28th Day	25	2.22	1.12	0.22
Air Test	Sodium fluoride	Baseline	25	2.08	0.70	0.14
		Immediate	25	0.94	0.56	0.11
		14th Day	25	0.48	0.47	0.09
		28th Day	25	0.34	0.43	0.09
	Acidulated phosphate fluoride	Baseline	25	1.96	1.01	0.20
		Immediate	25	1.46	0.90	0.18
		14th Day	25	1.28	0.97	0.19
		28th Day	25	1.14	0.96	0.19
	Propolis	Baseline	25	2.14	0.80	0.16
		Immediate	25	1.84	1.01	0.20
		14th Day	25	1.68	1.03	0.21
		28th Day	25	1.66	0.95	0.19

**Table 2:** Comparison of three clinical products (sodium fluoride, acidulated phosphate fluoride, and propolis) for dentin hypersensitivity by at different follow-up periods by two-way repeated measures ANOVA.

Assessment Method	Source	Type III Sum of Squares	df	Mean Square	F-Value	*p*-Value	Partial Eta Squared (Effect Size)
Tactile test	Groups	24.47	2	12.24	7.3240	0.0001 *	0.23
	Time	1300.88	3	433.63	363.5090	0.0001 *	0.94
	Groups with Time	16.62	6	2.77	9.9560	0.0001 *	0.29
	Total	1341.97	11	122.00			
Cold test	Groups	20.82	2	10.41	11.5620	0.0001 *	0.33
	Time	1310.46	3	436.82	425.3980	0.0001 *	0.95
	Groups with Time	9.69	6	1.61	6.7170	0.0001 *	0.22
	Total	1340.97	11	121.91			
Air test	Groups	38.13	2	19.06	20.0750	0.0001 *	0.46
	Time	46.78	3	15.59	112.0960	0.0001 *	0.82
	Groups with Time	13.27	6	2.21	15.4010	0.0001 *	0.39
	Total	98.18	11	8.93			

* Statistically significant at 5% level of significance.

**Table 3:** Pair-wise comparison with Bonferroni adjustment of three clinical products (sodium fluoride, acidulated phosphate fluoride, and propolis) for dentin hypersensitivity, assessed by tactile, cold, and air tests.

Assessment Method	(I) Groups	(J) Groups	Mean Difference (I–J)	Std. Error	*p*-Value	95% Confidence Interval for Difference	
						Lower Bound	Upper Bound
Tactile test	Sodium fluoride	Acidulated phosphate fluoride	−0.45	0.14	0.0110 *	−0.80	−0.09
	Sodium fluoride	Propolis	−0.69	0.18	0.0020 *	−1.14	−0.24
	Acidulated phosphate fluoride	Propolis	−0.25	0.22	0.8560	−0.82	0.33
Cold test	Sodium Fluoride	Acidulated phosphate fluoride	−0.54	0.12	0.0010 *	−0.84	−0.23
	Sodium fluoride	Propolis	−0.58	0.12	0.0001 *	−0.88	−0.28
	Acidulated phosphate fluoride	Propolis	−0.05	0.16	1.0000	−0.46	0.37
Air test	Sodium fluoride	Acidulated phosphate fluoride	−0.50	0.12	0.0010 *	−0.82	−0.18
	Sodium fluoride	Propolis	−0.87	0.13	0.0001 *	−1.20	−0.54
	Acidulated phosphate fluoride	Propolis	−0.37	0.16	0.0910	−0.78	0.04

* Statistically significant at 5% level of significance.

**Table 4:** Post hoc pairwise comparison of reduction in dentin hypersensitivity score among the products assessed with different assessment methods.

	Groups	Sodium Fluoride_Tactile	Sodium Fluoride_Cold	Sodium Fluoride_Air	Acidulated Phosphate Fluoride_Tactile	Acidulated Phosphate Fluoride_Cold	Acidulated Phosphate Fluoride_Air	Propolis_Tactile	Propolis_Cold	Propolis_Air
Pairs		*p*-Value	*p*-Value	*p*-Value	*p*-Value	*p*-Value	*p*-Value	*p*-Value	*p*-Value	*p*-Value
Baseline	Immediate	<0.001 *	<0.001 *	<0.001 *	<0.001 *	<0.001 *	0.003 *	<0.001 *	<0.001 *	0.019 *
	14th Day	<0.001 *	<0.001 *	<0.001 *	<0.001 *	<0.001 *	<0.001 *	<0.001 *	<0.001 *	0.002 *
	28th Day	<0.001 *	<0.001 *	<0.001 *	<0.001 *	<0.001 *	<0.001 *	<0.001 *	<0.001 *	<0.001 *
Immediate	14th Day	<0.001 *	<0.001 *	<0.001 *	<0.001 *	<0.001 *	1.000	<0.001 *	<0.001 *	0.176
	28th Day	<0.001 *	<0.001 *	<0.001 *	<0.001 *	<0.001 *	0.090	<0.001 *	<0.001 *	0.714
14th Day	28th Day	0.003 *	<0.001 *	0.033 *	<0.001 *	0.027 *	0.097	<0.001 *	0.368	1.000

* Statistically significant at 5% level of significance.

## Data Availability

Data can be made available for academic purposes upon request from the chief researcher via email.
